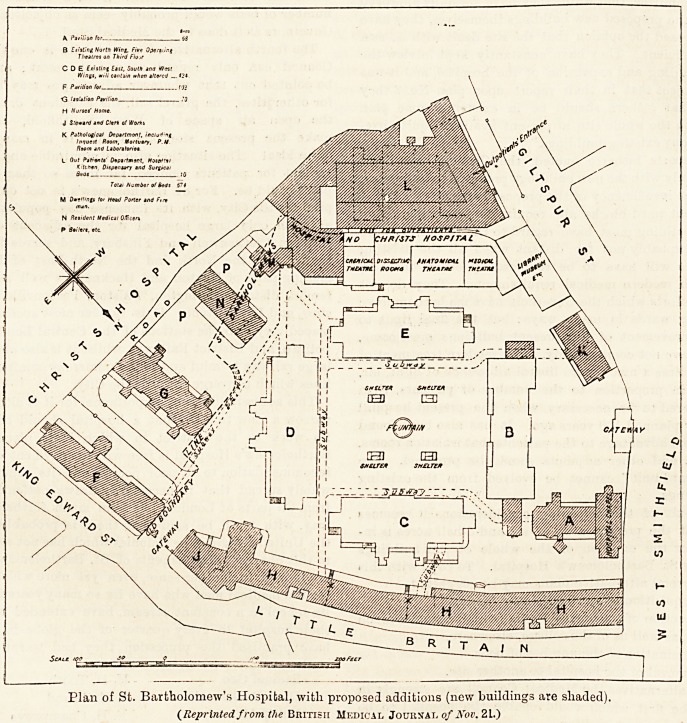# St. Bartholomew's Hospital

**Published:** 1903-11-28

**Authors:** 


### ST. BARTHOLOMEW'S HOSPITAL.

(*Reprinted from the* British Medical Journal *of Nov.* 21.)

The accompanying plan is a reproduction of that to which reference was made in the annotation on the subject of St. Bartholomew's Hospital in the *British Medical Journal* of November 14th. It is one of the several plans which the be obtained by adding a story to the north block. Superficially these advantages may appear considerable and the plan satisfactory, but to any one who considers the plan as a whole the question cannot but arise, "Is this to be the sole outcome of all the controversy which has taken place, and will this plan really result in a hospital which will thoroughly meet not only all the recognised requirements of the present day, but also anticipate those demands which can be foreseen as likely to be made by science in the near future?" In short, will this new hospital be not only one at which no one can throw a ready stone, but one which Londoners in the early future like their predecessors of the past can safely point out to foreigners as a building and institution thoroughly representative of British knowledge of the sciences of medicine and surgery?

Plan of St. Bartholomew's Hospital, with proposed additions (new buildings are shaded).
(*Reprinted from the* British Medical Journal *of Nov.* 21.)

Mansion House Committee had laid before it at its meetings in the spring, and it is also that which the Committee recommended for adoption to the Governors of the hospital.

From an examination of it, it is possible to gather a general idea of the alterations which it is proposed to effect, and of the way in which the acre and a half which has so far been acquired from Christ's Hospital is to be utilised. We pointed out some of its main features last week, and it will be noted that while it entails the sacrifice of the ancient church and the residential quarters, it provides a new out-patient department, a new nurses' home, new provision for isolation, and in addition five new operating theatres, to

We are afraid that the answer to this question must be unhesitatingly in the negative, and for sufficiently obvious reasons. The new out-patient department is undoubtedly good, but the general plan leaves, as we said last week, about 104 beds crowded on to each acre of the site, and two-thirds of these beds will be located in what now are, and have already long been, old-fashioned buildings. These main wards will, moreover, now have the added and great disadvantage of being surrounded by new buildings of greater or less elevation.

In fact, the faults of the plan are so obvious that we are entitled to inquire more particularly how it ever came to be propounded and upon what grounds the Mansion House Committee recommended it to the Governors of the hospital. Turning to the report of its proceedings issued by the former body we find that it definitely committed itself to two main conclusions, and somewhat less finitely to a third. The first of these was that in the public interest the hospital ought to be rebuilt where it is, and not, as had been suggested, moved elsewhere; the second, that the hospital had managed its affairs with economy and skill in the past, and was fully justified in making a great and exceptional appeal for assistance at the present crisis; thirdly, that rebuilding of the hospital was urgently needed, and there was ample room on the present site for the provision of a hospital with every modern appliance. A *précis* of the evidence upon which the first two conclusions were founded was supplied in the report, and they are not open to dispute; but no grounds for the latter conclusion were given, and, though the opinions of the medical and surgical staff on the alternative plans are quoted, nothing whatever is said as to any evidence having been given in support of the view that "there is ample room on the present site" for a perfect modern hospital.

We should be sorry to conclude after what has been stated, that the plan of rebuilding suggested really represents what the united wisdom of the Medical Committee of this great hospital regards as a perfect hospital, and we shall refuse to believe it until we have a definite and authoritative statement to that effect. What we can believe, however, is that the staff has simply given the plan a qualified approval, and that the plan represents, in short, a *pis aller* to which the staff has assented merely by way of making the best of a bad job when and because it was found that appeals to the governors to buy more ground were useless. Fragmentary statements which confirm this view have crept into the press, and some distinct evidence in support of it is supplied by a letter from Dr. Hensley, one of the consulting physicians.

Such evidence, however, is insufficient, and matters have reached such a pass that the medical staff of the hospital is bound in defence of its own reputation not less than for the sake of the general hospital interests of all London to state definitely, authoritatively, and collectively whether it is thoroughly satisfied with the plan put forward, or whether it considers more ground essential for the building of a proper hospital. If the answer to the former question is in the negative, the Governors are not justified in appealing to the public for support, and if they do do so they are not likely to receive it. If, on the other hand, they enlarge their scheme, and put forward one which they can show has the full and unqualified approval of the Medical Committee, they ought to, and we believe will be enabled by the public to put that plan into execution even if the expenditure entailed be a very much larger one than any that has yet been authoritatively suggested. We put this issue to the Medical Committee and to the Governors as one upon which the public has an absolute right to expect a definite reply, and we appeal to our contemporaries not to allow consideration of this important subject to be obscured any longer by the echo of past controversies and the discussion of by-issues.

## Figures and Tables

**Figure f1:**